# Effect of Ultrasonic Bending Vibration Introduced by the L-shaped Ultrasonic Rod on Solidification Structure and Segregation of Large 2A14 Ingots

**DOI:** 10.3390/ma13030807

**Published:** 2020-02-10

**Authors:** Chen Shi, Yongjun Wu, Daheng Mao, Gaofeng Fan

**Affiliations:** 1 College of Mechanical and Electrical Engineering, Central South University, Changsha 410083, China; llswyj@csu.edu.cn (Y.W.); fangaofeng@csu.edu.cn (G.F.); 2 State Key Laboratory of High Performance Complex Manufacturing, Central South University, Changsha 410083, China; 3 Light Alloy Research Institute, Central South University, Changsha 410083, China; mdh@csu.edu.cn

**Keywords:** L-shaped ultrasonic wave guide rod, ultrasonic bending vibration, 2A14 aluminum alloy, solidification structure, composition segregation

## Abstract

In order to achieve long-term and stable ultrasonic treatment in the direct chill semi-continuous casting process, a new L-shaped ceramic ultrasonic wave guide rod is designed to introduce ultrasonic bending vibration into 2A14 aluminum alloy melt. The effect of ultrasonic bending vibration on the solidification structure and composition segregation of large 2A14 aluminum alloy ingots (φ 830 mm × 6000 mm) in the process of semi-continuous casting were studied by means of a direct reading spectrometer, scanning electron microscope, metallographic microscope, and hardness test. The ultrasonic ingot treated by bending vibration was compared with the ingot without ultrasonic treatment and the ingot treated by the traditional straight-rod titanium alloy wave guide rod. The results show that, during the solidification of 2A14 aluminum alloy, ultrasonic treatment can significantly refine the grain, break up the agglomerated secondary phase, and make its distribution uniform. The macro-segregation degree of solute including the negative segregation at the edge of the ingots and the positive segregation in the center can be reduced. Through comparative analysis, the macrostructure of the ingot, treated by the L-shaped ceramic ultrasonic wave guide rod, was found to be better than that of the ingot treated by the traditional straight-rod titanium alloy wave guide rod. In particular, the grain refinement effect at the edge of the ingot was the best, the secondary phase was smaller, more solute elements can be dissolved into the α-Al matrix, and the ability of the L-shaped ultrasonic wave guide rod to restrain segregation was stronger at the edge of the ingot.

## 1. Introduction

A 2A14 aluminum alloy is a typical Al-Cu-Mg-Si alloy. Because of its high copper content, it has high strength and belongs to high strength duralumin [1,2,3]. It has good heat resistance, forging and welding properties, and it can be manufactured into free forgings, and die forgings with complex shapes, which are widely used in the field of aerospace [4,5,6,7,8]. Direct chill semi-continuous casting is one of the most effective technologies to produce large aluminum alloy ingots [9]. Due to the uneven distribution of temperature field and flow field in the solidification areas during casting, there are many problems in the large-scale aluminum alloy ingots, produced by traditional casting methods, which can easily cause an uneven distribution of solidification structure and solute composition, and defects, such as coarse grain, composition segregation, hot cracking, and shrinkage cavities. In particular, macro-segregation directly affects the quality of ingots and the subsequent finished products ratio of forging, and increases the material processing loss and production cost [10,11,12,13].

At present, applying appropriate ultrasonic vibration in the process of metal solidification is a good method to reducing casting defects, obtaining good structure and improving mechanical properties of materials [14,15,16,17]. Abramov V et al. [18] studied the effect of the ultrasonic treatment of the water-cooled transducer on the microstructure and properties of different industrial aluminum based alloy. The effect of ultrasonic treatment on the microstructure of as-cast alloy can be summarized as follows: Reduction of mean grain size, variation of phase distribution, and better material homogeneity and segregation control. Eskin et al. [19] introduced ultrasonics into the semi-continuous casting of aluminum alloy, produced AA2324 aluminum alloy round ingot with a diameter of 1200 mm. It was found that the grain after ultrasonic treatment was generally refined, and the solidification structure was non-dendrite. Li Xiaoqian et al. [20,21,22,23] studied the principle and mechanism of ultrasonic treatment with the straight-rod wave guide rod, analyzed the influence of ultrasonic power, frequency, and insertion depth of ultrasonic rods on the quality of large-diameter ingot, carried out a large number of casting experiments, and successfully obtained 2XXX and 7XXX ultrasonic large-diameter aluminum alloy ingots of various sizes.

However, there are some problems in the ultrasonic treatment process, which hinder its wide application in metallurgical industry. The traditional ultrasonic radiation rod, made of titanium alloy, was seriously corroded in the molten metal, thereby, reducing its service life and polluting the aluminum melt [24,25,26]. Moreover, the ultrasonic transducer, connected with the traditional straight-rod titanium alloy wave guide rod, is directly above the high-temperature metal melt, and the melt produces direct high-temperature heat radiation to the ultrasonic transducer. This often leads to the detuning problem of an ultrasonic vibration system or even damage of the transducer during the casting process, so it is difficult to achieve long-term stable ultrasonic treatment [27,28]. In this work, to overcome these problems, a new L-shaped ceramic ultrasonic wave guide rod was designed to introduce ultrasonic bending vibration into 2A14 aluminum alloy melt. The differences between treatment by the L-shaped ultrasonic wave guide rod and treatment by the traditional straight-rod titanium alloy wave guide rod were compared from the aspects of macrostructure, micro-grain size and morphology, secondary phase characteristics and composition segregation. The purpose of this paper is to verify the effectiveness of applying the L-shaped ultrasonic wave guide rod on the preparation of homogeneous large-scale aluminum alloy ingots from the experimental point of view, and then to find out the optimal ultrasonic introduction process, in order to provide certain theoretical guidance for industrial production.

## 2. Material and Methods

### 2.1. Experimental Equipment

**Figure 1:**
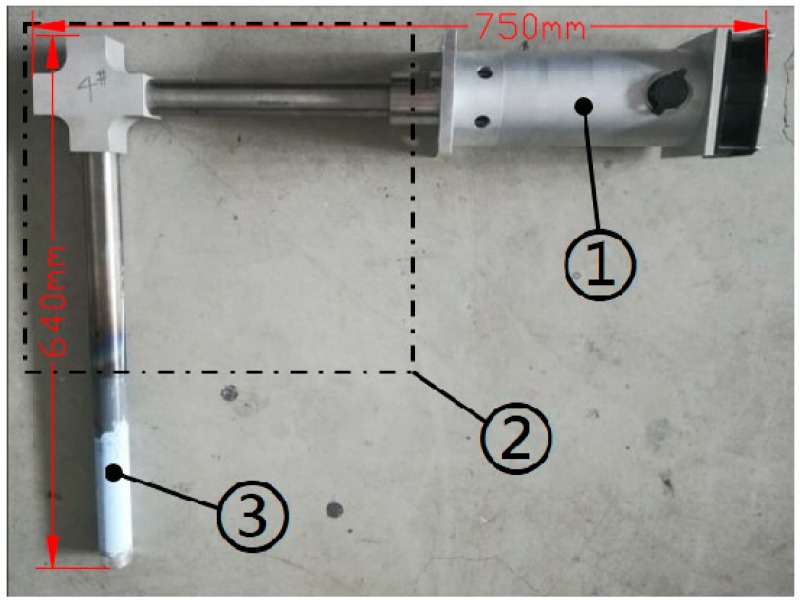
Structure of ultrasonic guided wave device.

A new type of ultrasonic guided wave device which mainly composed of (1) a magnetosteric transformer, (2) a L-shaped solid horn and (3) a tool head was designed, as shown in the Figure 1. The working frequency of ultrasonic was 19 ± 0.2 kHz, the power variation range was 0–1000 W, and the amplitude was 10 μm. The L-shaped horn was composed of the first level horn, which propagated the longitudinal vibration horizontally, and the second level horn, which propagated the bending vibration vertically. The first level horn and the second level horn, as well as the tool head, transducer and L-shaped horn were all connected by thread. The material of the horn was TC4 titanium alloy, and the material of the tool head in direct contact with the metal melt was nano-silicon nitride ceramics. The experimental device adopted the L-shaped ultrasonic wave guide rod, which made the ultrasonic transducer not directly above the high-temperature molten metal, and effectively avoided the direct radiation of high-temperature heat flow to the ultrasonic transducer, so as to improve the working stability and reliability of the ultrasonic vibration system. Moreover, the L-shaped solid horn transformed the sine wave transmitted longitudinally into the distorted wave transmitted transversely, so that the tool head inserted into the metal melt had strong ultrasonic transmission on its end face and side, thus strengthening the mechanical vibration and acoustic streaming of ultrasonic in the melt.

### 2.2. Materials

2A14 aluminum alloy is an aluminum alloy with Cu, Mg, and Si as the main alloying elements. The experimental material was the 2A14 aluminum alloy with the chemical compositions listed in Table 1.

### 2.3. Experimental Process

**Figure 2:**
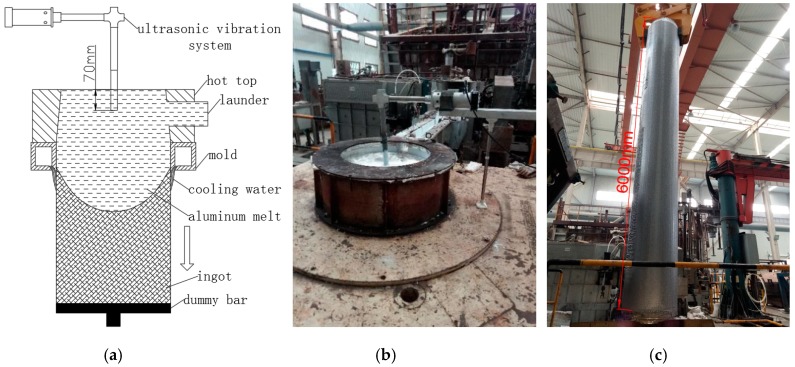
2A14 aluminum alloy ultrasonic semi-continuous casting: (**a**) schematic diagram of casting; (**b**) photo of casting site; and (**c**) ingot product.

The technological process of this ultrasonic casting experiment was as follows: The first step was to bake the smelting furnace, and then about 10 tons of commercial purity aluminum were put into the furnace. The material was heated and melted, and then fully stirred by electromagnetic stirrer. After the surface scum of the aluminum liquid was removed, metal additives, like Cu and Mg, as well as master alloy were added into the aluminum melt. Then, the sample was sent to the analysis room for composition detection and analysis. After the compositions of the molten aluminum alloy reached the required standard, Al-Ti-B wire rod was added. The molten aluminum was drained into the hot top mold through the launder, and the casting started after the hydrogen measurement of the melt was qualified. As shown in Figure 2a,b, ultrasonic wave was introduced into the aluminum melt from the top directly above the center to realize ultrasonic casting. Finally, the large ingot, as shown in Figure 2c, was transferred to the soaking pit furnace for homogenization treatment for 72 h. After annealing treatment, the chill layer of about 10 mm thick on the surface of ingots was removed by lathe. The specific casting parameters are shown in Table 2. In order to evaluate the effect of ultrasonic guided wave device ultrasonic treatment, the specific experimental conditions and variables were set as follows: (1) no ultrasonic treatment; (2) the pre-heated L-shaped ultrasonic wave guide rod was immersed 70 mm below the liquid level of the melt, and the ultrasonic treatment with a power of 500 W was applied; (3) the pre-heated the straight-rod wave guide rod was immersed 70 mm below the liquid level of the melt, and the ultrasonic treatment with a power of 500 W was applied.

**Figure 3:**
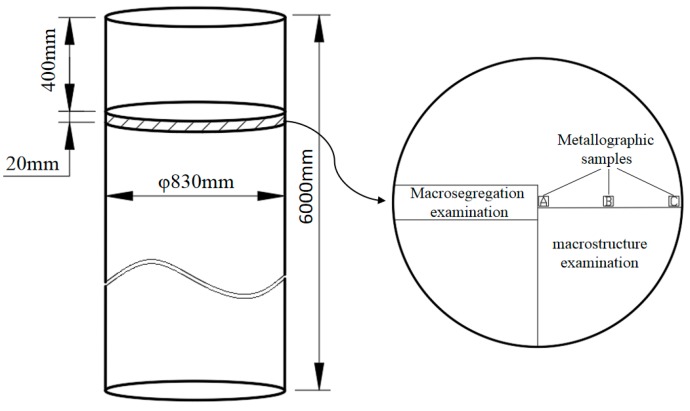
Sampling locations of ingot samples.

A circular section with a thickness of 20 mm was cut at 400 mm away from the head of ingots with a vertical band saw. A quarter of the section was taken for macrostructure analysis, and a long strip with a width of 100 mm was cut along the radius direction for spectral analysis. The sampling locations is shown in Figure 3. The specific analysis contents were as follows:

Firstly, the macrostructure of the samples was analyzed. After milling, the samples were polished with fine sandpaper, then the surface was cleaned with anhydrous ethanol, and the surface was treated with Keller’s solution for 10–20 s to observe the corrosion effect. Then, the strip sample was milled, and the compositions were detected along the radius direction by the SPECTRO MAXx direct reading spectrometer. A group of data were measured every 40 mm. Each group of data measured three points and took the average value of them. Finally, the distribution curve of the alloy elements in the ingots along the radial direction was drawn by using the measured data. Moreover, the microstructure analysis was carried out. The sampling positions are shown in Figure 3. The positions A, B, and C in the figure correspond to the structure of the center, the one half radius, and the edge of the ingot samples, respectively. After the selected samples were polished, they were wiped with a cotton ball. The secondary phase structure and energy spectrum analysis were carried out using a Phenom fully automatic scanning electron microscope (Phenom-world BV, Holland). Then, their Vickers hardness values were measured using a HV-1000A microhardness tester. The applied load was 1 N with the dwell time of 10 s. Five results were tested for every samples, and after the maximum and minimum values were discarded, an average of the remaining three values was applied. Finally, the metallographic microstructure of the samples corroded by Keller’s solution was observed using the OLYMPUS DSX500 metallurgical microscope (OLYMPUS Corporation, Japan), and the photos were taken, and the grain sizes were measured by the OLYMPUS Stream image analysis software. The three-circle intercept procedure was adopted. The measuring grid consists of three concentric circles of equal distance. Their total perimeter is 500 mm. This grid was used to measure at least five different fields of view of any choice for each specimen. The intercept points were counted manually with a mouse, and the average of the line intercept was calculated automatically by the analysis software.

## 3. Results and Discussions

### 3.1. Effect on Macrostructure

**Figure 4:**
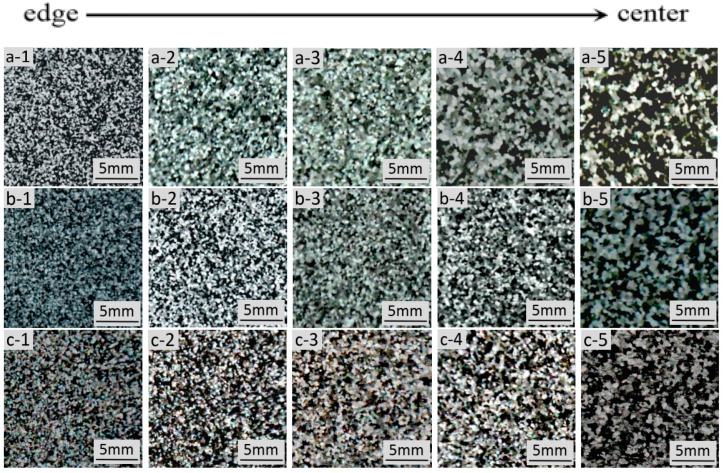
Macrostructure of obtained under different processing conditions: (**a**) no ultrasonic treatment; (**b**) treatment by the L-shaped ultrasonic wave guide rod; (**c**) treatment by the straight-rod ultrasonic wave guide rod.

Figure 4 shows the macrostructure of the three casting ingots under different casting processes. Figure 4a shows that there are many dendrites, and coarse grains in the macrostructure of the ingot, without ultrasonic treatment. By comparing the images from the a-1 to the a-5 of the Figure, it was found that the grain near the edge of the ingot is the smallest, and the grain size from the edge to the center of the ingot is increasing. This is because the direct effect of cooling water, near the edge of the mold, made the under-cooling of the aluminum liquid in the edge area increase sharply, which induced homogeneous nucleation, thus, forming a large amount of crystal nuclei and fine equiaxed grains. However, in the center of the ingot, higher melt temperature produce coarser grains, mostly because of the de-activation of potent solidification sites. Comparing Figure 4a–c demonstrated that the macrostructure of the ingots with ultrasonic treatment is smaller and more uniform, compared with the ingot without ultrasonic treatment. Further comparison between Figure 4b and c shows that the effect of treatment by the L-shaped ultrasonic wave guide rod is slightly better than that of the traditional straight-rod wave guide rod on the whole. The grain size of the macrostructure of the ingots with ultrasonic treatment is smaller than that of the ingot without ultrasonic treatment, but the difference between them is not very obvious. This is mainly because a large amount of Al-Ti-B wire rod were added to the aluminum melt, as grain refiners during semi-continuous casting, which had achieved good grain refinement effect. Overall, the grain refinement effect under the synergistic effect of ultrasonic and grain refiners were significantly better than under by adding only grain refiners. The treatment by the L-shaped ultrasonic wave guide rod can not only significantly improve the refining ability of Al-Ti-B refiner, but also expand the effective range of the refiners.

### 3.2. Effect on Microstructure

**Figure 5:**
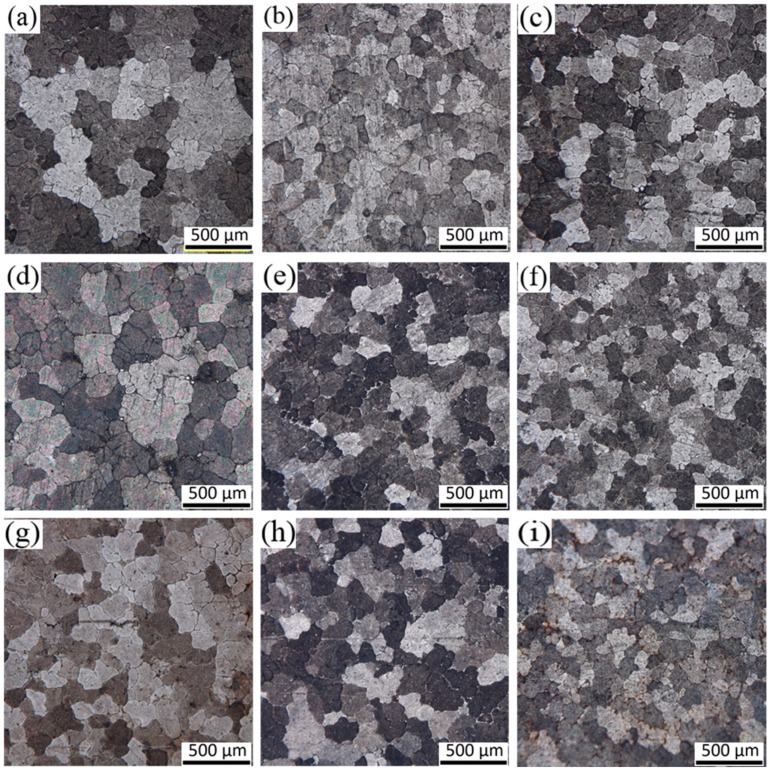
Microstructure of obtained under different processing conditions: (**a**) Center of ingot without ultrasonic; (**b**) 1/2 radius of ingot without ultrasonic; (**c**) edge of ingot without ultrasonic; (**d**) center of ingot by L-shaped ultrasonic; (**e**) 1/2 radius of ingot by L-shaped ultrasonic; (**f**) edge of ingot by L-shaped ultrasonic; (**g**) center of ingot by straight-rod ultrasonic; (**h**) 1/2 radius of ingot by straight-rod ultrasonic; and (**i**) edge of ingot by straight-rod ultrasonic.

**Figure 6:**
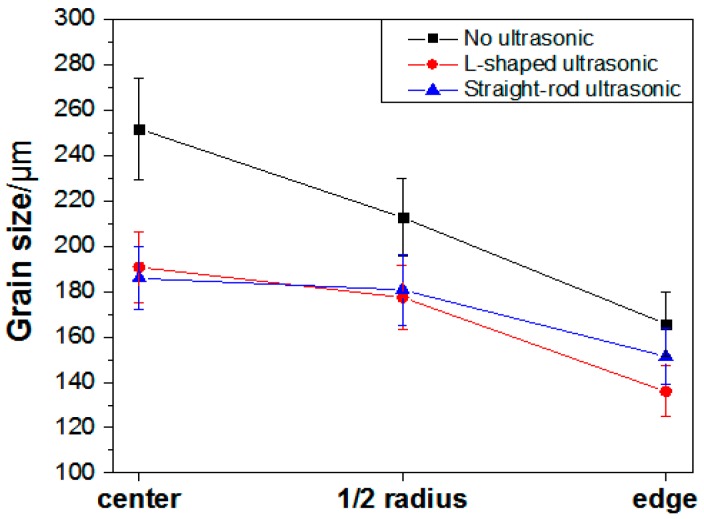
Distribution of grain size of ingots under different processing conditions.

Figure 5 shows the microstructure of the ingots cross-section along the radius under different processing conditions. It can be seen from the figure that the grain size of the three ingots is decreasing from the center to the edge. The dendrites in the center of the conventional ingot are dominant, the secondary dendrite arms are large, and only a few fine grains exist. At the one half radius mark, the number of coarse dendrites decreases and the grain size is smaller. The grain is basically fine equiaxed at the edge. After ultrasonic treatment, the grain of the ingots is refined to a certain extent, mainly fine equiaxed grain with more uniform distribution, especially in the center and at the 1/2 radius. Obviously, the closer the distance from the ultrasonic tool head, the better the grain refinement effect, which shows that the effect of grain refinement of ultrasonic treatment is closely related to its range of action. From the distribution curve of grain size of the ingots, under different processing conditions in Figure 6, it can be seen that in the conventional ingot, the grain size in the center is the thickest with an average grain size of 251.74 μm. Under the action of the L-shaped ultrasonic wave guide rod and the straight-rod ultrasonic wave guide rod, the average grain sizes decreased to 190.62, and 185.81 μm, respectively, and the decreasing amplitude exceeded 60 μm. At the edge, the average grain size of the ingot treated by the L-shaped ultrasonic wave guide rod is 135.96 μm, and the refining effect is better than that of the ingot treated by the straight-rod ultrasonic wave guide rod. The mechanism of ultrasonic grain refinement can be attributed to the cavitation effect and acoustic steam of ultrasonic [29]. The L-shaped ultrasonic wave guide rod had end radiation, but also more side radiation, compared with the action of the straight-rod ultrasonic wave guide rod, which made the area of ultrasonic cavitation larger. The shock wave generated in the process of cavitation bubbles collapse had a strong crushing effect on the primary dendrite and growing dendrite structure in the aluminum alloy melt, which increased the number of crystal nucleus during the solidification process, and the cavitation effect led to instantaneous local lower cold, in part, and heterogeneous nucleation. At the same time, the instantaneous high-pressure, produced by the collapse and fracture of the cavitation bubbles, constantly impacted the surface of heterogeneous particles in the melt, increased the contact angle with aluminum liquid, improved its wettability, activated the heterogeneous particles, and promoted the nucleation core to increase during solidification and crystallization. A large amount of nuclei was generated in these areas, thereby, increasing the number of nucleation and refining microstructure [23]. There was still a certain sound flow effect at the edge of the ingots, which made the aluminum melt be stirred continuously and forced the grain to disperse evenly in the melt. At the same time, it made the temperature field and solute field more uniform in the mold, and the growth direction of the grain became relatively uniform, so as to achieve the effect of refining grain.

### 3.3. Effect on the Secondary Phase

**Figure 7:**
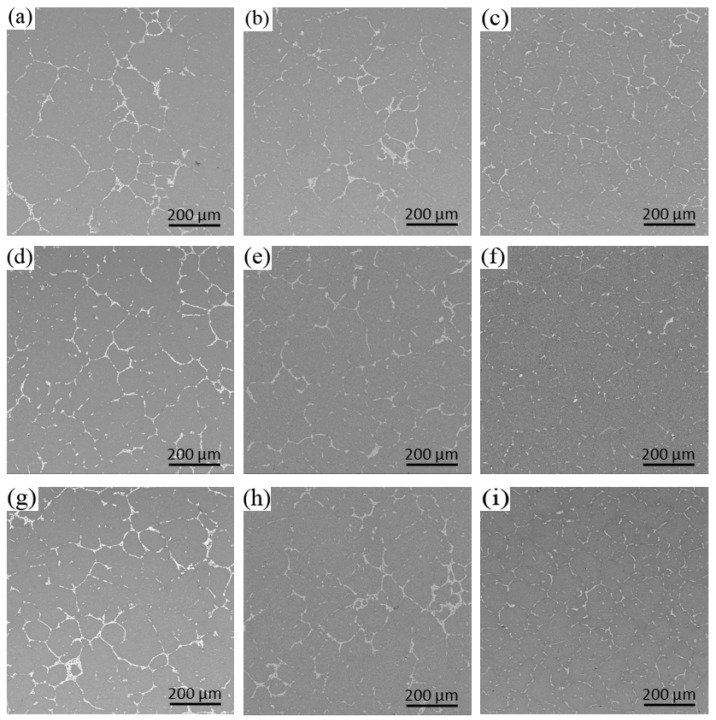
Distribution of the secondary phase of the ingots under different processing conditions: (**a**) Center of ingot without ultrasonic; (**b**) 1/2 radius of ingot without ultrasonic; (**c**) edge of ingot without ultrasonic; (**d**) center of ingot by L-shaped ultrasonic; (**e**) 1/2 radius of ingot by L-shaped ultrasonic; (**f**) edge of ingot by L-shaped ultrasonic; (**g**) center of ingot by straight-rod ultrasonic; (**h**) 1/2 radius of ingot by straight-rod ultrasonic; and (**i**) edge of ingot by straight-rod ultrasonic.

Figure 7 shows the distribution of the secondary phase along the radial direction of the ingots under different processing conditions. The dark gray area is α-Al matrix, and the white part is the secondary phase. In the solidification process of the alloy, the matrix phase formed first, and then the alloy elements precipitated, due to the decrease in solubility of the matrix, forming binary or ternary eutectic phase structure with Al element. It can be seen from the figure that the grain size in the microstructure of the conventional ingot is obviously larger, and there are some dendrites, while the proportion of equiaxed grain in the ingots by ultrasonic treatment is larger, the grain becomes smaller and more uniform, the size of the secondary phase is also reduced, the morphology is improved, and the distribution is more uniform. All of these significantly improved the comprehensive mechanical properties of the ingots. In the center of the ingots, whether it is the conventional ingot, without ultrasonic treatment, or the ingots with ultrasonic treatment, the secondary phase agglomeration phenomenon at the grain boundaries is very obvious and there is still serious segregation. At the one half radius of the ingots, the ultrasonic treatment broke-up some of the clustered secondary phase, and branched intergranular secondary phase with a discontinuous distribution appeared. In particular, the crushing effect of the ingot, treated with the L-shaped ultrasonic wave guide rod, was found to be better than that of ingots treated with the straight-rod ultrasonic wave guide rod, and the coarse secondary phase on the grain boundary was reduced, but there are still obvious long strip and massive eutectic phases. At the edge of the ingots, there are relatively few agglomerations of the secondary phase, and the secondary phase is mainly in the form of fine mesh. The secondary phase of the ingot, treated by the straight-rod wave guide rod, is increasingly fine, distributed in an intermittent network at the grain boundary of equiaxed grain, and has been basically dissolved in the matrix in some areas.

**Figure 8:**
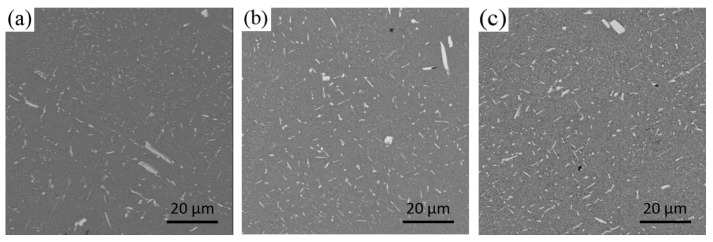
Distribution of the secondary phase in the grain at the 1/2 radius: (**a**) No ultrasonic treatment; (**b**) treatment by the L-shaped ultrasonic wave guide rod; (**c**) treatment by the straight-rod ultrasonic wave guide rod.

**Figure 9:**
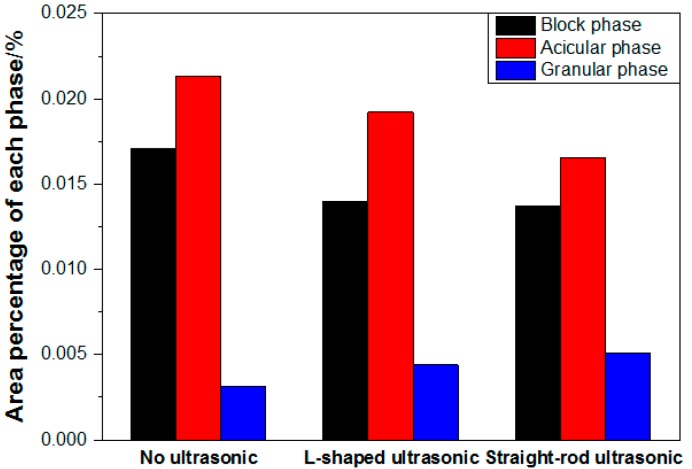
Area percentage of each secondary phase in the grain at the 1/2 radius.

Figure 8 and Figure 9 show the distribution and the area percentage of the secondary phase in the grain at the 1/2 radius respectively. The coarse bars and blocks of the secondary phase in the conventional ingot without ultrasonic treatment have larger proportion and are dispersed in the grain. The secondary phase in the grain of the ingots by ultrasonic treatment is mainly granular and acicular, and the distribution is relatively uniform, but there are still a great deal of little block or short rod-like secondary phase. The application of ultrasonic treatment can refine the secondary phase in the grain, break and re-melt the primary dendrite in the solidification front of the melt, reduce its aggregation and growth, and promote its uniform distribution.

**Figure 10:**
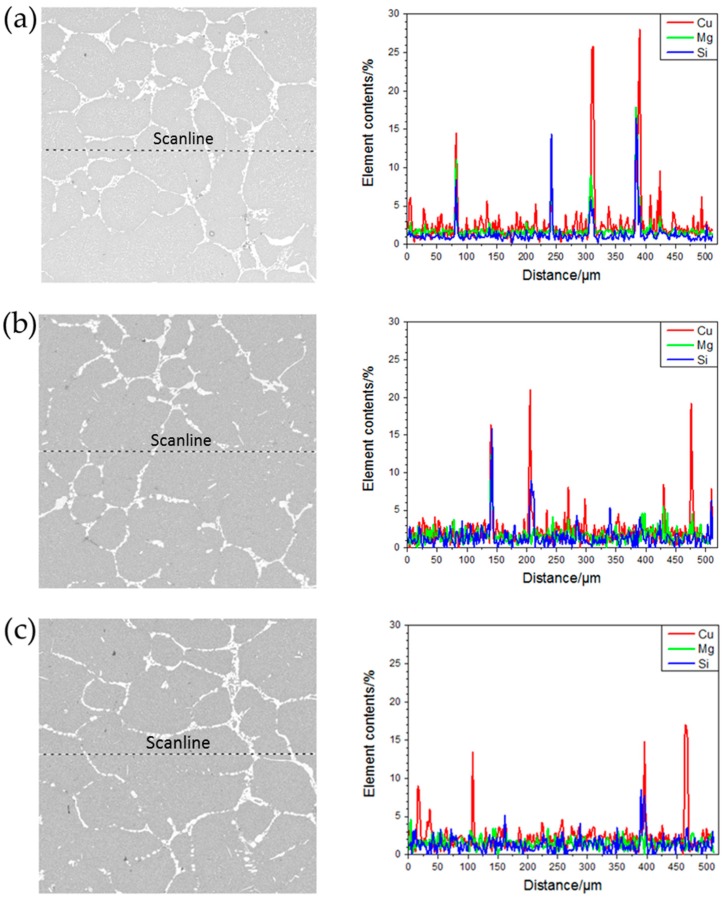
Results and areas of line scan analysis of main alloy elements in the ingots under different processing conditions: (**a**) No ultrasonic treatment; (**b**) treatment by the L-shaped ultrasonic wave guide rod; and (**c**) treatment by the straight-rod ultrasonic wave guide rod.

Figure 10 shows the results of line scan analysis of main alloy elements in the ingots under different processing conditions. It can be seen from the figure that the main alloy elements Cu, Mg, and Si, in the ingots without ultrasonic treatment, have obvious segregation at the grain boundary, and the segregation of Cu element is particularly serious. Also, Cu, Mg, and Si are mainly enriched in the secondary phase, Al_2_Cu, which is white. In the ingots treated by ultrasonic, the concentration of main alloy elements decreased, solute diffusion was promoted, part of Al_2_Cu phase dissolved in the matrix, but a large amount number of Cu element were still segregated at the grain boundary.

Further point scanning analysis was carried out on the α-Al matrix structure of the ingots under different processing conditions. About 100 points in one grain were selected for scanning, and the average value of the measured 100 component points was taken as the main content of solute elements in the matrix. Table 3 shows the content of solute elements in the α-Al matrix structure of the ingots treated with different processing conditions.

### 3.4. Effect on MacroSegregation

The segregation rate ΔC was calculated by the following equation,

(1) $$\Delta C=\frac{C_{i}-C_{0}}{C_{0}}$$

where $C_{i}$ is the mean composition at a specific location, $C_{0}$ is the average (or nominal) alloy composition. ΔC > 0 represents positive segregation while ΔC < 0 represents negative segregation. The segregation index S is introduced to measure the overall macrosegregation degree of ingots in radial direction,

S = Δ*C*_max_ − Δ*C*_min_ (2)

where Δ*C*_max_ and Δ*C*_min_ are the maximum, and minimum, segregation rates in the selected direction, respectively. The larger the S value is, the greater the macrosegregation degree of elements is, and the more uneven the solute distribution is.

**Figure 11:**
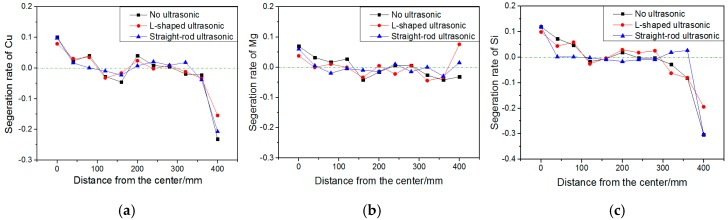
Variation of segregation rate of Cu (**a**), Mg (**b**), and Si (**c**) elements in the cross section of ingots under different processing conditions.

Figure 11 shows the variation of segregation rate of Cu, Mg, and Si elements in the cross-section of the ingots under different conditions, which can clearly and intuitively reflect the macrosegregation of the ingots. The ingot without ultrasonic treatment has a large positive segregation in the center, and a very serious negative segregation near the edge, and the composition distribution curve has a sharp drop in the melt at the position. The segregation index of Cu, Mg, and Si decreases from 0.078, 0.053 and 0.072 to 0.048, 0.038 and 0.055, respectively after ultrasonic treatment of the L-shaped ultrasonic wave guide rod within 100 mm from the center. The segregation index of Cu, Mg, and Si decreases from 0.086, 0.069, and 0.036 to 0.044, 0.025, and 0.014, respectively after ultrasonic treatment of the straight-rod ultrasonic wave guide rod within 100 mm at the 1/2 radius. The segregation index of Cu, Mg, and Si changes from 0.212, 0.016 and 0.276 to 0.143, 0.120 and 0.132, respectively after ultrasonic treatment of the L-shaped ultrasonic wave guide rod near the edge. The application of ultrasonic treatment can effectively reduce the degree of solute segregation, reduce the negative segregation at the edge of ingots, reduce the positive segregation in the center, and make the distribution of solute elements in different parts of ingots tend to be uniform. However, the effect of the ultrasound is still limited, and the distribution of Cu and Si elements in the surface and center areas is still uneven, and there is a large gap between the two areas. The ability of the L-shaped ultrasonic wave guide rod in restraining segregation is stronger in the side of ingots, and the ability of the straight-rod ultrasonic wave guide rod to restrain segregation is stronger in the center and at the one-half radius of ingots. This is mainly because the L-shaped ultrasonic wave guide rod had not only the end radiation, but also more side radiation compared with the action of the straight-rod ultrasonic wave guide rod. Especially, micro jet with high velocity was formed on the side of the ultrasonic rod, which forced the local melt in the mold to produce convection in the radial direction and played a certain role in stirring the aluminum melt. Stirring reduced the solidification speeds at the edge of the ingots to a certain extent, so that the components in the solidification process had more time to diffuse, thus, greatly unifying the solute field, reducing the concentration gradient at the solidification front, as well as the concentration of solute elements. Under the action of strong shock wave and micro jet produced by cavitation effect, the high solute liquid at the solidification front was rapidly diffused, thus reducing the macro negative segregation at the edge of the ingots [30]. However, due to the rapid attenuation of ultrasound in the liquid, the effect of ultrasonic at the edge of the mold was very weak, compared with that of the center. Although, it can improve the inverse segregation of solute elements to a certain extent, it cannot be eliminated fundamentally, and the effect is limited. During the solidification process, the positive segregation on the surface of ingots occurred, while the negative segregation near the surface was caused by the deformation of dendrite network [31]. In the surface area of the ingots, cooling water was directly sprayed on it, the cooling rate was high, and the temperature gradient in solidified shell was very large. The thermal shrinkage and volume deformation of the dendrite network at the edge of ingots were larger than the internal area of ingots, and the resulting liquid-phase flow towards the ingot surface was also very obvious, while, the intergranular melt flowed continuously in the direction of shrinkage. When the ingot moved to the lower part of the mold, the solidification latent heat also made the rigid mushy zone on the ingot surface partially re-melt. The melt rich in solute elements between dendrites moved to the shell of the ingots along the gaps driven by extrusion pressure, which resulted in poor solute area near the surface of ingots and negative segregation. However, the solute elements were highly enriched on the surface of the ingots, and the composition was significantly higher than the average value, forming obvious segregation tumor, which reduced the quality of ingot surface.

### 3.5. Effect on Mechanical Property

**Figure 12:**
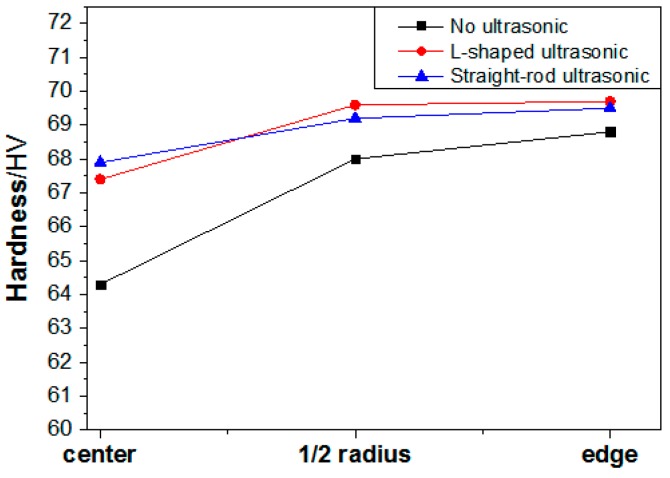
Hardness test results along the radial direction of the ingots under different processing conditions.

Hardness is a comprehensive index to measure the strength, plasticity, fracture toughness, deformation resistance, and other mechanical properties of aluminum alloy. Figure 12 shows the hardness test results, along the radial direction of the ingots, under different processing conditions. It can be seen from the figure that the hardness values of every position of the ingots, with ultrasonic treatment, is higher than that of the ingot without ultrasonic treatment, and the values tend to be consistent. The hardness values rose greatly in the center of ingots treated with the L-shaped ultrasonic wave guide rod and the straight-rod ultrasonic wave guide rod, increasing by 4.8%, and 5.6%, respectively. Previous studies have shown that, when ultrasonic treatment is applied to the solidification process of the aluminum alloy, the cavitation effect and acoustic steam increase the nucleation rate, refine the grain, and make the solidification structure more compact and uniform. At the same time, porosities, inclusions, and other defects are also effectively suppressed, and the volume fraction and size of the secondary phase is reduced, the morphology and distribution of the secondary phase are improve, and the composition segregation can also be restrained to a certain extent. The comprehensive effect of these factors effectively improved the mechanical properties of ultrasonic ingots. Compared with other positions, the effect of grain refinement in the center of ingots was better, the secondary phase was smaller and more evenly distributed, and the hardness of aluminum alloy was greatly improved. At the edge of the ingots, the fine equiaxed crystal structure increased the resistance of dislocation movement, thus, increasing the strength. However, due to the existence of serious negative segregation, the decrease of Cu content resulted in the decrease of the amount of Al_2_Cu in the matrix, and the hardness values of aluminum alloy were not much higher than that at the 1/2 radius of the ingots. The mechanical properties and isotropy of the ingots, treated by ultrasonic, improved, which is beneficial to the subsequent processing.

## 4. Conclusions

(1) The macrostructure of the ingots, with ultrasonic treatment, is smaller and more uniform. The L-shaped ultrasonic wave guide rod had not only the end radiation, but also more side radiation, compared with the action of the straight-rod ultrasonic wave guide rod and the effect of treatment, is better at the edge.

(2) Ultrasonic treatment can break the agglomerated secondary phase, and the secondary phase of the ingots treated by the L-shaped ultrasonic wave guide rod is smaller, and more solute elements are dissolved into the α-Al matrix.

(3) Ultrasonic treatment can effectively decrease the degree of solute segregation. The ability of the L-shaped ultrasonic wave guide rod to restrain segregation is stronger at the edge of ingots, and the ability of the straight-rod ultrasonic wave guide rod to restrain segregation is stronger in the center and at half a radius of ingots.

The effect is good in applying the L-shaped ultrasonic wave guide rod on the preparation of homogeneous large-scale aluminum alloy ingots, which will make it possible that the new L-shaped ceramic ultrasonic wave guide rod can be widely used in metallurgical industry. In our future research, the mechanical properties of the ingots, the resistance to high-temperature metal melt erosion and service life of the ultrasonic rod, and the working stability of the ultrasonic system, will be explored.

## Figures and Tables

**Table 1:** Chemical compositions of the 2A14 aluminum alloy (wt.%).

Element	Si	Cu	Mg	Zn	Mn	Ti	Ni	Fe	Al
Content	0.6–1.2	3.9–4.8	0.4–0.8	≤0.30	0.4–1.0	≤0.15	≤0.10	0.0–0.7	Bal.

**Table 2:** Casting process parameters of the 2A14 aluminum ingots.

Size/mm	Pouring Temperature/°C	Cooling Temperature/°C	Speed of Water Flow/(L/min)	Speed of Introducing Ingot/(mm/min)
φ 830 × 6000	706	24	540	22

**Table 3:** Content of solute elements in the α-Al matrix structure of the ingots treated under different processing conditions (Wt.%).

Solute Elements	No Ultrasonic	L-Shaped Ultrasonic	Straight-Rod Ultrasonic
Cu	3.40	3.65	3.68
Si	0.89	1.21	1.17
Mg	0.94	1.07	1.09
